# Supplementary material for: MetaRibo-Seq measures translation in microbiomes
Source: Nat Commun. 2020 Jun 29;11:3268. doi: 10.1038/s41467-020-17081-z (PMC7324362; doi:10.1038/s41467-020-17081-z)
Supplement: Supplementary file 10 — Supplementary Data 7 [file 41467_2020_17081_MOESM10_ESM.zip › File2/Confidence_High_Taxonomy/178261_out.krona.html]

Javascript must be enabled to view this page.

members
magnitude
magnitudeUnassigned
count
unassigned
taxon
rank

178261\_out

37

37
superkingdom
2

37
phylum
976

class
200643
37

37
171549
order

family
815
37

816
genus
37

species

SRS011134\_contig\_number\_52435SRS013687\_contig\_number\_22286SRS016095\_contig\_number\_18842SRS016989\_contig\_number\_10415SRS017859\_contig\_number\_5567SRS018575\_contig\_number\_7486SRS020328\_contig\_number\_5177SRS021153\_contig\_number\_3493SRS021219\_contig\_number\_6720SRS022137\_contig\_number\_12796SRS022713\_contig\_number\_1962SRS023914\_contig\_number\_2709SRS024549\_contig\_number\_4252SRS042966\_contig\_number\_19566SRS045713\_contig\_number\_8265SRS053214\_contig\_number\_1573SRS054905\_contig\_number\_9439SRS055982\_contig\_number\_1842SRS056519\_contig\_number\_10937SRS075021\_contig\_number\_2814SRS075878\_contig\_number\_22917SRS077194\_contig\_number\_19174SRS077730\_contig\_number\_328SRS077849\_contig\_number\_13238SRS097889\_contig\_number\_17459SRS1041090\_contig\_number\_6505SRS1041112\_contig\_number\_2044SRS1041130\_contig\_number\_1238SRS1041136\_contig\_number\_27034SRS1041139\_contig\_number\_1944SRS104636\_contig\_number\_25254SRS104975\_contig\_number\_20819SRS1055022\_contig\_number\_10789SRS142503\_contig\_number\_28980SRS143148\_contig\_number\_23804SRS143780\_contig\_number\_11536SRS147977\_contig\_number\_879
818
37
